# Crosstalk between salicylic acid signalling and the circadian clock promotes an effective immune response in plants

**DOI:** 10.1038/s44323-024-00006-0

**Published:** 2024-09-02

**Authors:** Olivia J. P. Fraser, Samantha J. Cargill, Steven H. Spoel, Gerben van Ooijen

**Affiliations:** The University of Edinburgh, School of Biological Sciences, Max Born Crescent, EH9 3BF Edinburgh, UK

**Keywords:** Plant sciences, Diseases

## Abstract

The rotation of Earth creates a cycle of day and night, leading to predictable changes in environmental conditions. The circadian clock synchronizes an organism with these environmental changes and alters their physiology in anticipation. Prediction of the probable timing of pathogen infection enables plants to prime their immune system without wasting resources or sacrificing growth. Here, we explore the relationship between the immune hormone salicylic acid (SA), and the circadian clock in Arabidopsis. We found that SA altered circadian rhythmicity through the SA receptor and master transcriptional coactivator, NPR1. Reciprocally, the circadian clock gates SA-induced transcript levels of NPR1-dependent immune genes. Furthermore, the clock gene *CCA1* is essential for SA-induced immunity to the major bacterial plant pathogen *Pseudomonas syringae*. These results build upon existing studies of the relationship between the circadian clock and SA signalling and how interactions between these systems produce an effective immune response. Understanding how and why the immune response in plants is linked to the circadian clock is crucial in working towards improved crop productivity.

## Introduction

The rotation of our planet creates 24-h cycles in our environments, such as in light levels and temperature. To anticipate these rhythms, the circadian clock has evolved as an internal timekeeping mechanism in almost all eukaryotes. It synchronizes an organism’s behavioural and physiological processes with the rhythmic changes in its surroundings^1,2^. The temporal separation or coordination of process that results from this timekeeping enables the optimization of resource use in an organism’s everyday function^3,4^.

As sessile organisms, plants cannot escape unfavourable conditions, evade herbivores, or move to find more distant resources. Therefore, the main challenge for plants is the effective use of available resources to improve fitness. Plants utilize the circadian clock to track and anticipate periodic environmental changes and stressors. This results in the prioritizing of distinct biological responses at different times of day or year according to the anticipated environmental conditions^4–7^.

In Arabidopsis, circadian rhythms involve an interconnected network of transcriptional/translational feedback loops, in which circadian components activate or repress each other’s activity in a cyclical manner^7^. The core of the circadian clock consist of the morning expressed MYB-related transcription factors *CIRCADIAN CLOCK ASSOCIATED 1 (CCA1*) and *LATE ELONGATED HYPOCOTYL (LHY*) and the evening expressed *TIMING OF CAB EXPRESSION 1 (TOC1*). *CCA1, LHY* and *TOC1* negatively regulate each other’s expression alongside other components of the clock^8–12^. The rhythmic oscillations of *CCA1, LHY* and *TOC1* regulate many downstream pathways through periodic gene expression. An estimated 30% of the *Arabidopsis thaliana* (*Arabidopsis*) transcriptome is under circadian control^13^. This includes genes involved in regulating flowering, biomass, photosynthesis, water use, temperature stress responses and pathogen defences^4,14,15^.

One of the downstream pathways that the clock modulates is the immune system^16^. Plant pathogens and pests cause devastating losses to food crops worldwide^17^. Understanding the mechanisms that underpin plant immunity is critical to improving crop productivity and to meet the exponentially increasing food demand. Pathogen recognition is conferred through two immune pathways: PAMP triggered immunity (PTI) and effector triggered immunity (ETI)^18,19^. Pathogen recognition by these interconnected pathways leads to accumulation of the immune hormone salicylic acid (SA) that activates the expression of thousands of immune genes, many via the activation of the master transcriptional coactivator, NPR1^20,21^. NPR1 is responsible for the regulation of pathogenesis related (PR) genes including *PR1*. In addition to activating local immune responses, SA and NPR1 are also required for the onset of systemic acquired resistance (SAR), which provides long-lasting protection throughout the entire plant against a broad spectrum of pathogens^22–25^.

Basal levels of SA are under circadian control with SA abundance peaking in the middle of the night, which is thought to prime the immune response for dawn, when plants are most vulnerable to pathogen infection^26–28^. In accordance, *Arabidopsis* displays enhanced resistance in the subjective morning and increased susceptibility at subjective midnight^29^. This disparate resistance is lost in plants overexpressing *CCA1* (*CCA1ox*) and in *elf3-1* mutants, both of which are arrhythmic at the gene expression levels. Furthermore, key immune genes are under circadian regulation, including the pathogen recognition receptors *FLS2* and *EFR*, as well as components of the SA biosynthesis, *ICS1* and *EDS1*^29^. Interestingly, rhythmic levels ofis an SA receptor and master regulator the active NPR1 monomer have also been observed^30^.

There is also evidence for reciprocal modulation of the circadian clock by the immune system, however the nature of clock alteration and the extent to which it is modified are unclear. *Arabidopsis* infected with *Pseudomonas* or treated with the bacterial elicitor flg22 display a significantly shortened circadian period^31^. The current research is not in agreement regarding the effect of SA on circadian clock rhythms. Zhou et al.^30^ found that SA treatment caused an increase in the amplitude of *TOC1* but not *CCA1* rhythms in an NPR1-dependent manner. On the other hand, SA treatment has also been reported to cause a significant reduction of amplitude and delay in circadian phase^32^. Furthermore, Philippou et al.^33^ indicate that SA treatment shortens the period of *CCA1* and *TOC1* rhythms in the absence of sucrose.

Taken together, these findings point to extensive crosstalk between the immune system and the circadian clock. However, the exact mechanisms and extent of cross modulation are poorly understood.

In this study, we further explore the relationship between SA-dependent immune signalling and the circadian clock in *Arabidopsis*. We aimed to establish the role of NPR1 in the link between SA-signalling and circadian clock rhythms and investigate the potential involvement of core clock genes *CCA1* and *TOC1* in the modulation between the two systems. Indeed, our study reveals the critical role of NPR1 in the SA-induced modulation of *CCA1* and *TOC1* rhythms. We also establish the importance of clock gene *CCA1* in launching an effective SA-induced immune response, demonstrated in both SA-induced *PR1* transcript levels and SA-induced resistance to *Pseudomonas*.

## Results

### SA treatment shortens the period of the circadian clock

Conflicting reports exist on the effects of SA on the circadian clock in plants. Therefore, we first aimed to consolidate the effect of SA on the clock within our experimental systems and conditions. Leaf discs of plants expressing firefly luciferase (LUC) driven by the rhythmic promoters of either *CCA1* or *TOC1* (*CCA1pro:LUC* and *TOC1pro:LUC*, respectively) were subjected to a range of SA concentrations.

**Fig. 1:**
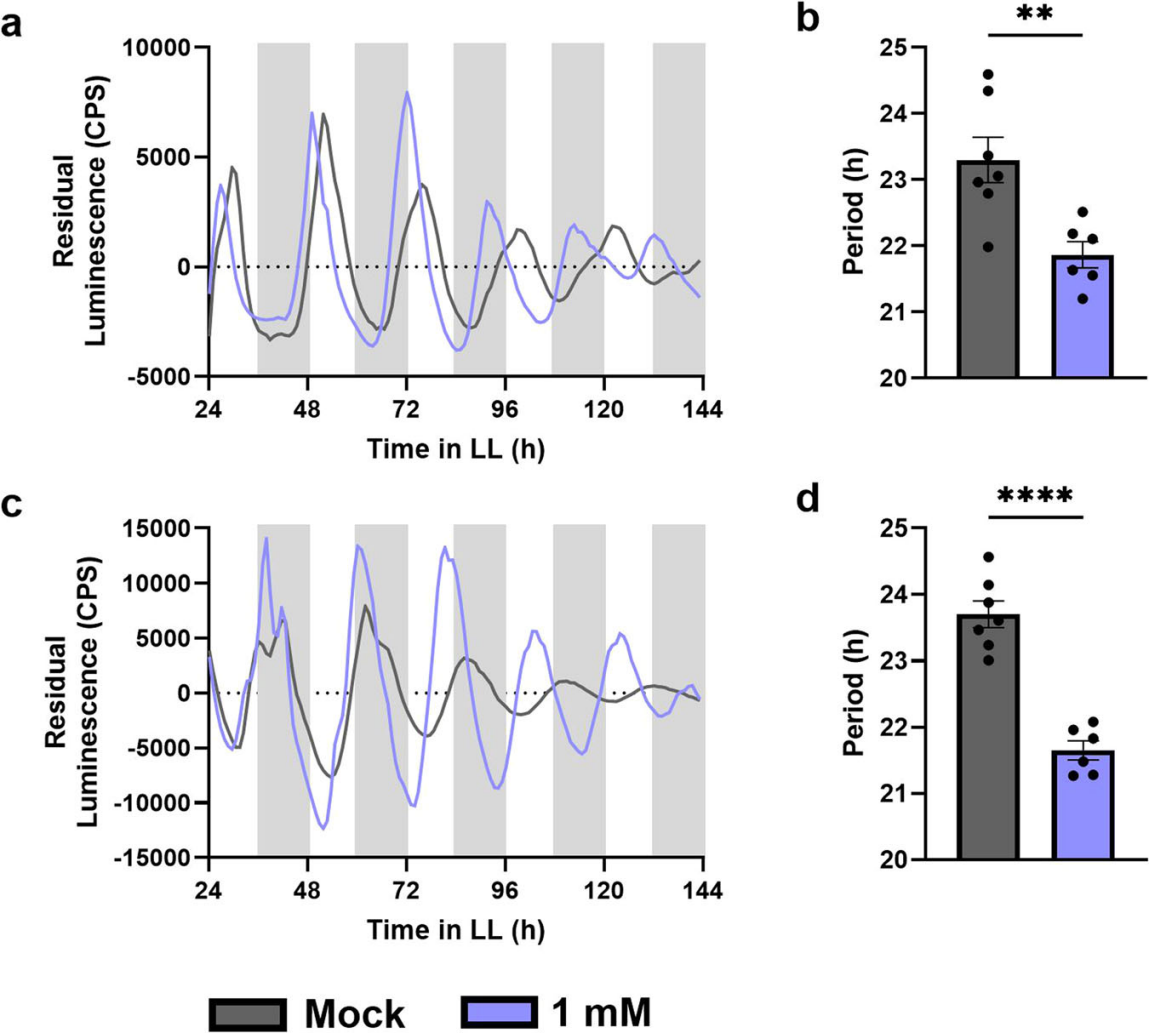
Continuous SA treatment shortens the period of the circadian clock. Promoter activity of *CCA1* (**a**, **b**) and *TOC1* (**c**, **d**) was observed by measuring luminescence in *CCA1pro:LUC* and *TOC1pro:LUC* leaf disks, respectively. Leaf disks were treated with 1 mM SA (blue) or mock treated with water (grey). The mean period (**b**, **d**) was calculated from the 24–120 hr time window. An unpaired *t* test was performed between each mock and treated data set for *CCA1* and *TOC1*: *p < 0.05, **p < 0.01, ***p < 0.001, ****p < 0.0001, unpaired t-test. Error bars indicate mean ± SEM (*n* = 7). Data are from a single experiment representative of 3 independent repeats.

Treatments with 100 µM SA (Supplementary Fig. 2) or 1 mM SA (Fig. 1 and Supplementary Fig. 1) shortened the periods of both *CCA1* and *TOC1* under constant light conditions. Higher concentrations of SA abolished rhythms while lower concentrations did not significantly alter rhythms (Supplementary Fig. 2). The raw luminescence data indicate that, though the period-shortening effect is consistent, there is not a consistent effect of 1 mM SA treatment on amplitude (Supplementary Fig. 1).

Thus far, we have observed the effects of continuous SA treatment on clock rhythms, however, as pathogen-induced SA accumulation is transient^34^, we next investigated the effect of transient SA treatment on *CCA1* rhythms. *CCA1pro:LUC* leaf disks were treated with SA at 5 different time points over 24 h. The media was replaced with SA-free imaging media 8 h post-treatment. Only time points LL34 and LL44 exhibited significant period shortening (Supplementary Fig. 3). We observed no effect on phase or amplitude.

Overall, these results indicate that exogenous SA treatment modulates the circadian clock in a dose-dependent manner.

### SA-induced period shortening is dependent on NPR1

Our results suggest that SA treatment alters circadian clock rhythms in *Arabidopsis*. NPR1 is an SA receptor and master regulator of SA-responsive immune gene expression^20^. Zhou et al.^30^ previously reported that the effect of SA on *TOC1* is dependent on NPR1. Contrasting results reported by Li et al.^32^ argue that NPR1 antagonizes the clock response to SA treatment. As NPR1 is a regulator of SA-dependent immunity, it may mediate feedback between SA and the clock. However, in previous studies that observed circadian gene expression of NPR1 mutants, no effect on period was found^30,32^.

**Fig. 2:**
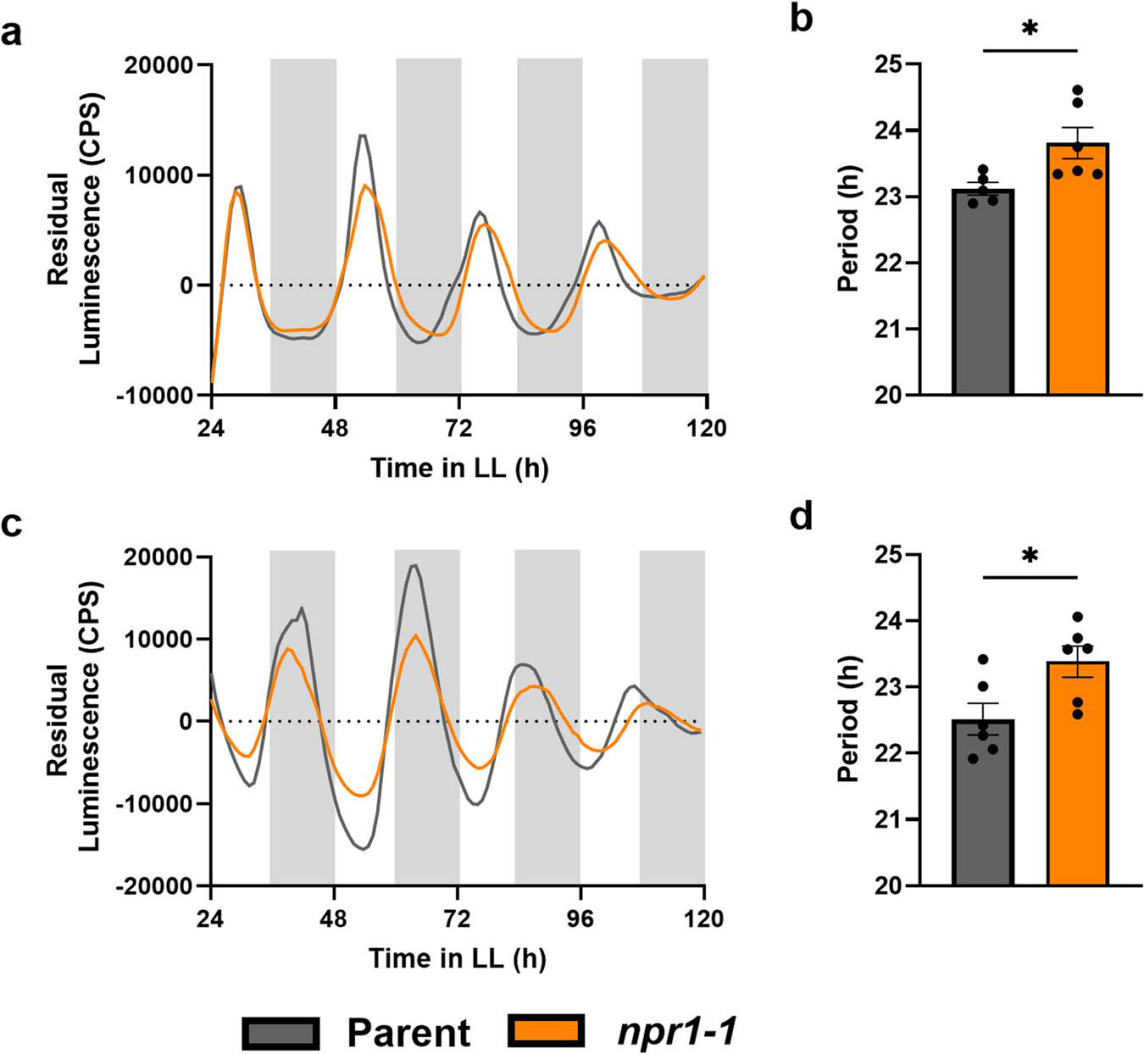
The circadian clock of *npr1-1* displays a longer period than wild type. Promoter activity of *CCA1* (**a**, **b**) or *TOC1* (**c**, **d**) was measured in the parent line parent line (grey) and the *npr1-1* mutant (orange). *CCA1* promoter activity (**a**, **b**) was visualized by measuring luminescence of *CCA1pro:LUC* in wild-type (grey) versus *CCA1pro:LUC* in *npr1-1* (orange) leaf disks. *TOC1* promoter activity (**c**, **d**) was visualized by measuring luminescence of *TOC1pro:LUC* in wild-type (grey) versus *TOC1pro:LUC* in *npr1-1* (orange) leaf disks. The mean period was calculated from the 24–120 h time window from traces of *CCA1* (**b**) and *TOC1* (**d**): *p < 0.05, **p < 0.01, ***p < 0.001, ****p < 0.0001, unpaired *t* test. Error bars represent mean ± SEM (*n* = 6). The data shown here are from a single experiment representative of 3 independent repeats.

To investigate this, we observed *CCA1* and *TOC1* rhythms in *npr1-1* mutants. We crossed *CCA1pro:LUC* and *TOC1pro:LUC* with *npr1-1* mutants. First, we compared the rhythms of *CCA1* and *TOC1* in wild-type (WT) versus *npr1-1* mutant background (Fig. 2). We found that *npr1-1* mutants had a significantly longer period as observed for both *CCA1* (Fig. 2a, b) and *TOC1* (Fig. 2c, d). Although the period lengthening effect is small, the detailed time resolution provided by luciferase reporters allowed us to evidence a clear effect of NPR1 on circadian gene expression in uninduced conditions

**Fig. 3:**
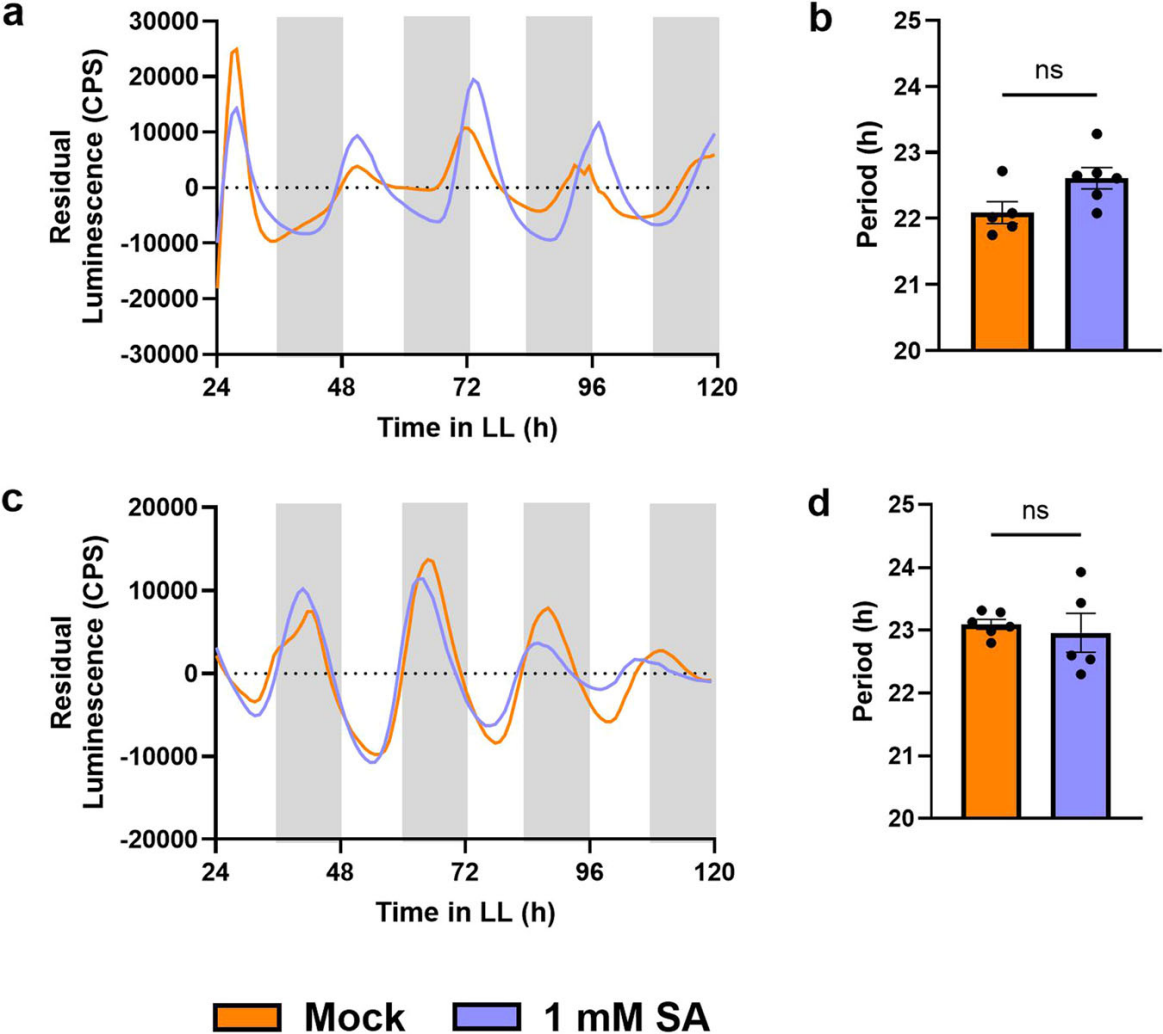
Period shortening by SA is lost in *npr1-1* plants. Promoter activity of *CCA1* (**a**, **b**) or *TOC1* (**c**, **d**) was observed by measuring luminescence in *CCA1pro:LUC* in *npr1-1* and *TOC1pro:LUC* in *npr1-1* leaf disks, respectively. Leaf disks were treated with 1 mM SA (blue) or mock treated with water (orange). The mean period was calculated from the 24–120 h time window from traces of *CCA1* (**b**) or *TOC1* (**d**): *p < 0.05, **p < 0.01, ***p < 0.001, ****p < 0.0001, unpaired *t* test. Error bars indicate mean ± SEM (*n* = 6). The data shown here are from a single experiment representative of 2 independent repeats.

To test if NPR1 also mediates the effects of SA on circadian period shortening (Fig. 1), we subjected our clock marker lines in the WT and *npr1-1* backgrounds to SA treatment. As opposed to the WT background (Fig. 1), SA treatment of *CCA1pro:LUC* in *npr1-1* and *TOC1pro:LUC* in *npr1-1* leaf discs did not alter rhythms (Fig. 3). Combined, these data demonstrate that NPR1 affects timekeeping and that the effect of SA on the circadian clock is dependent on NPR1. Thus, basal and SA-induced NPR1 are part of the input system of the plant circadian clock.

### SA-induced PR1 transcript levels are gated by the circadian clock

**Fig. 4:**
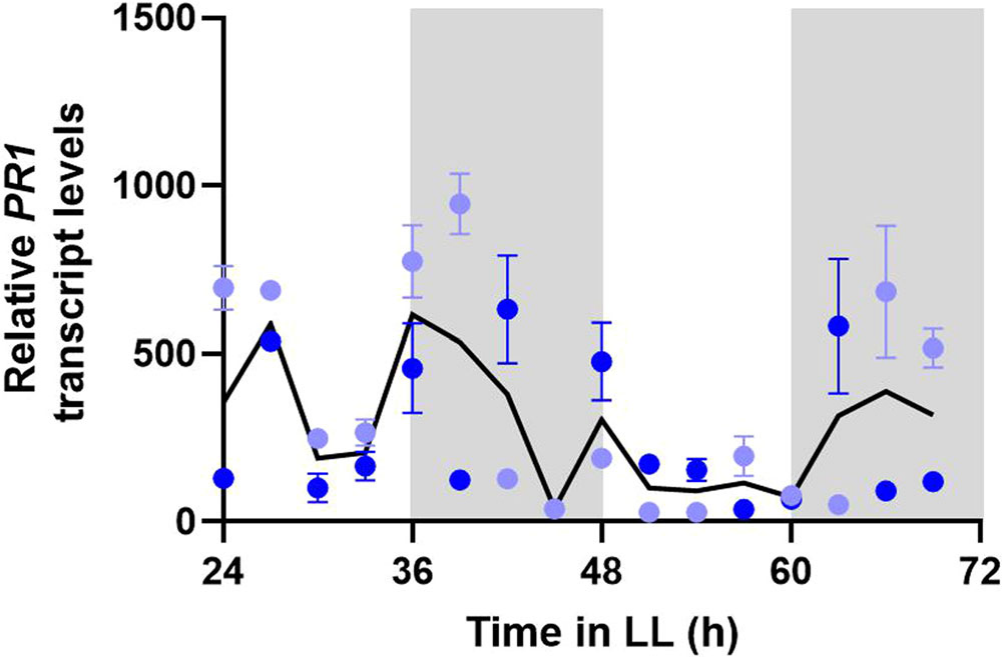
SA induced *PR1* transcript levels are higher during the subjective night. *Col-0* seedlings were grown on MS plates under 12/12 light/dark conditions for 14 days before being released into constant light (LL). Every 3 h, plates were sprayed with 1 mM SA. Relative transcript levels of PR1 were measured 6 h post-treatment. The data points are the mean ± SEM (*n* = 3) of quantitative PCR results from two biological replicates (light blue versus dark blue). The black line represents the mean values of the two biological replicates.

Thus far, NPR1 appears to be one of the key elements in crosstalk between SA levels and the circadian clock. NPR1 is a master regulator of SA-responsive gene expression, including the antimicrobial gene *PATHOGENESIS RELATED 1* (*PR1*). As endogenous basal levels of SA as well as the abundance of monomeric NPR1 protein exhibit circadian oscillations^28,30^, we investigated if *PR1* transcript levels are also rhythmic. We measured *PR1* transcript levels in wild-type plants by sampling every 3 h for 2 days. In the absence of an immune inducer, basal *PR1* transcript levels remained very low and did not exhibit rhythmicity (Supplementary Fig. 4). However, when we subjected plants to SA at 3-h intervals, we found that SA-induced *PR1* transcript levels were consistently higher during the subjective night than the subjective day (Fig. 4). Rhythmicity analysis of the mean SA-induced *PR1* transcript level of both biological replicates combined was assessed using the eJTK algorithm^35^. This analysis revealed that SA-induced *PR1* transcript levels are not rhythmic (*p* < 0.05) Thus, the circadian clock appears to gate SA-induced *PR1* transcript levels, though it is not rhythmic.

**Fig. 5:**
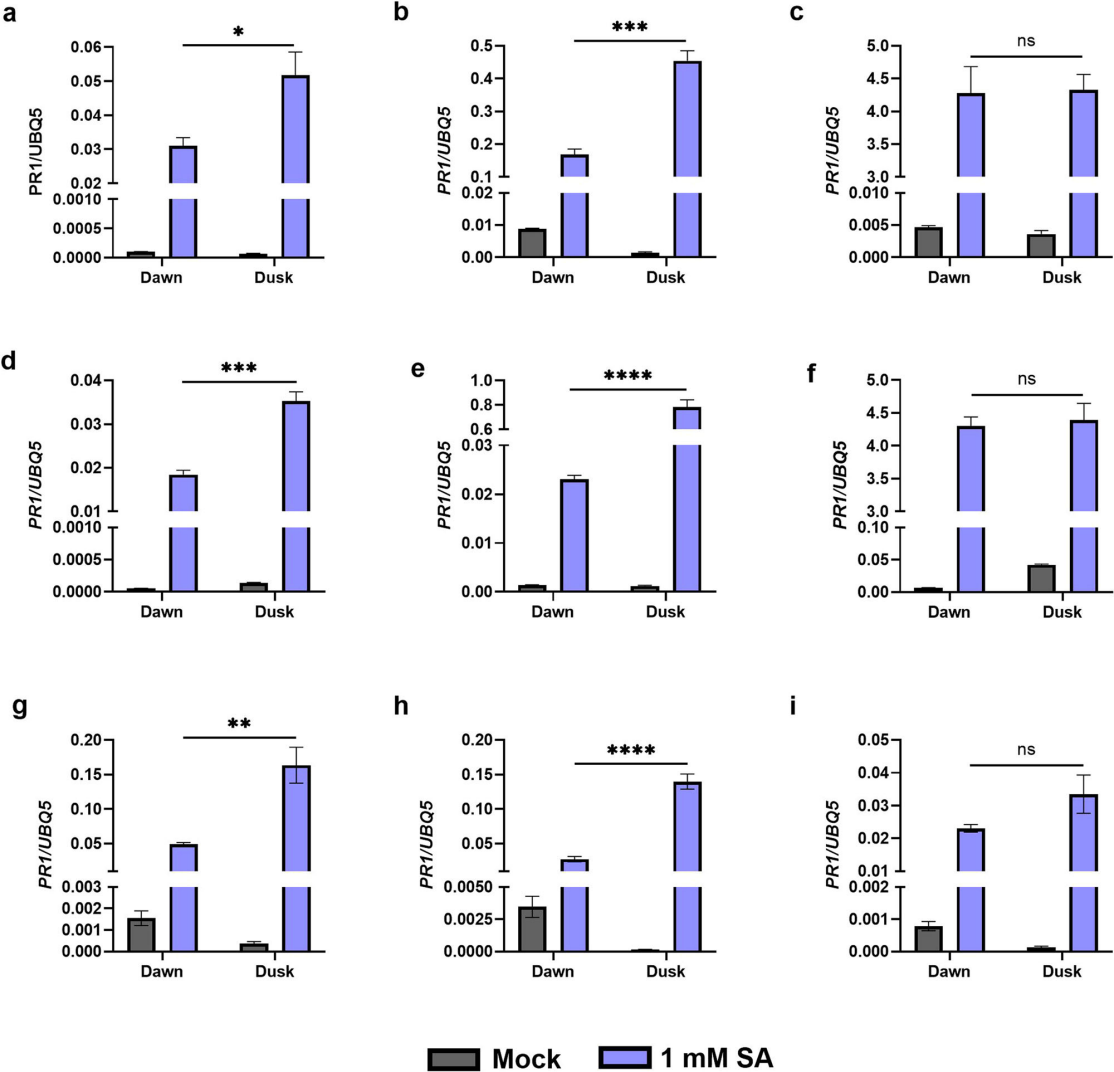
*CCA1* expression is required for the disparate transcript levels of *PR1* in response to SA treatment. *Col-0* (**a**, **d**, **g**), *CCA1ox* (**b**, **e**, **h**) and *cca1lhy* (**c**, **f**, **i**) seedlings were sprayed with 1 mM SA or mock (water) at subjective dawn versus dusk. Seedlings were sampled 6 h after treatment and *PR1*transcript levels were measured. *p < 0.05, **p < 0.01, ***p < 0.001, ****p < 0.0001, unpaired *t* test. Error bars indicate mean ± SEM (*n* = 4). The data shown here are from three independent experiments: experiment 1 (**a**–**c**); experiment 2 (**d**–**f**); experiment 3 (**g**–**i**). We present all the results to demonstrate consistency between the independent repeats.

To investigate this further, we compared SA-induced *PR1* transcript levels at subjective dawn versus dusk in wild-type as well as in arrhythmic *CCA1ox* plants^36^ and *cca1lhy* mutants that exhibit a shortened period^31^. As expected, wild-type plants exhibited stronger induction of *PR1* transcript levels in response to SA treatment at dusk (Fig. 5a, d, g). The difference in SA-induced *PR1* transcript levels between dusk and dawn was increased in *CCA1ox* plants (Fig. 5b, e, h), but completely lost in the *cca1lhy* mutant (Fig. 5c, f, i). These data indicate that core clock component *CCA1* gates SA-dependent transcript levels of *PR1* and enhances responsiveness to SA at dusk.

### CCA1 is required for SA-induced resistance

**Fig. 6:**
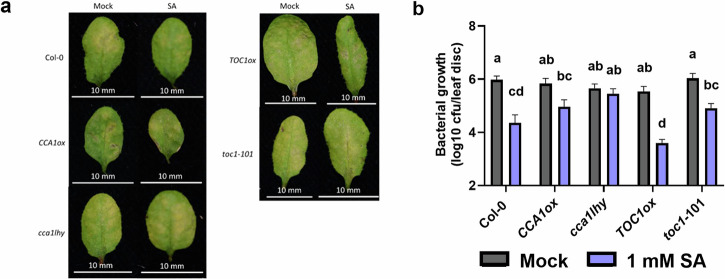
*CCA1* circadian mutants do not display SA induced resistance to *Pseudomonas.* *Col-0*, *CCA1ox, cca1lhy, TOC1ox* and *toc1-101* plants were grown under 12/12 light/dark conditions for 3 weeks. At dawn on day 21 (LL0), the plants were released into constant light conditions. At LL24 the plants were sprayed with 1 mM SA or mock (water). At LL48 the plants were infected with *Pseudomonas syringae pv. maculicola (Psm)*. The level of bacterial growth was measured 3 days post infection. **a** Leaves representative of the visual median level of infection at 3 days post-infection for each genotype and treatment. **b** The infection level at 3 days post-infection expressed by colony forming units (CFU, *y*-axis is logarithmic) in leaf disk extracts. Error bars represent mean ± SEM (*n* = 8 individual leaves). A two-way ANOVA and Tukey’s HSD Test were preformed to analyse the differences in bacterial growth between treatments and genotypes. The letters indicate significant differences between groups (*p* < 0.05). The data shown here are from a single experiment, representative of 3 independent experiments (*Col-0* versus *CCA1ox, cca1lhy* or *TOC1ox* [Supplementary Fig. 5a–c]) or 2 independent experiments (*Col-0* versus *toc1-101* [Supplementary Fig. 5c, d]).

Our results to this point demonstrate evidence of crosstalk between SA signalling and the circadian clock. In spite of the evidence for clock regulation of SA levels and immune gene expression^27–29^, no direct evidence exists for circadian involvement in the process of SA-induced resistance to pathogens. Therefore, we investigated the role of the circadian clock in establishing SA-induced resistance. We sprayed wild-type (*Col-0*), *CCA1ox*, *cca1lhy*, *TOC1ox* (arrhythmic^37^) and *toc1-101* (short period^38^) plants with SA at subjective dawn to mimic pathogen infection, followed 24 h later by infiltration with *Pseudomonas syringae pv. maculicola (Psm)* ES4326. We have displayed all the results for this assay (Fig. 6 and Supplementary Fig. 5) as the quantification of bacterial growth can be highly variable. As expected, wild-type plants displayed a significantly lower level of infection after treatment with SA (Fig. 6 and Supplementary Fig. 5). *TOC1ox* plants and *toc1-101* mutants also displayed SA-induced resistance, indicating that neither *TOC1* expression nor its rhythmicity is required for SA-mediated resistance. However, SA-induced resistance was not observed in *CCA1ox* plants or in *cca1lhy* mutants. These results indicate that both *CCA1* expression as well as its rhythmic expression are required to establish SA-dependent disease resistance. These results further evidence the critical role for *CCA1* in linking the circadian clock, SA signalling, and immunity.

## Discussion

Plant pathogens are responsible for large reductions in agricultural productivity^17^. A functioning circadian clock has been shown to enhance resistance to pests and pathogens both pre- and post-harvest^27,39^. The regulation of the immune system by the circadian clock has long been theorized to prime the immune system to the time of day that pathogen attack is most likely, thereby conserving the plants resources^27^. The alteration of circadian rhythms during infection may function to prioritize resource allocation towards an immune response by resetting the circadian clock to a time of day that facilitates this through its regulation of other plant processes.

The results presented in this study indicate that the immune hormone SA modulates that circadian clock, inducing NPR1-dependent period shortening. Previous studies have reported conflicting results regarding the effect of SA on circadian clock rhythms. Zhang et al.^31^ found that treating seedlings with the SA mimic benzo(1,2,3)thiadiazole-7-carbothioic acid (BTH) had no effect on *CCA1* promoter rhythms. In a similar study, Zhou et al.^30^ found that treatment with SA increased the amplitude of *TOC1* rhythms but not *CCA1* rhythms in 3-week-old Arabidopsis.

Li et al.^32^ monitored the effect of transient (4 h) SA treatment on clock gene rhythms, observing a significant reduction in amplitude and a delay in phase for *CCA1*, *TOC1* and *LHY* rhythms. This delay in phase was larger when SA treatment began closer to subjective dawn, though no such pattern was observed in the amplitude reduction. In contrast, when we applied SA transiently (8 h), period shortening of *CCA1* rhythms occurred at LL34 and LL44, but no effect on phase or amplitude was observed.

None of the studies mentioned above observed any effect of long-term SA exposure on period. However, Philippou et al.^33^ observed SA-induced period shortening of *GI* (*GIGANTEA*), *CCA1*, *CCR2* and *TOC1* rhythms, which was attenuated by the addition of sucrose. Therefore, the discrepancies between our study and the previous studies following SA treatment could be due to differences in the state of the plant material when monitored, such as the presence of sucrose, whole plant versus leaf disk or potentially light levels. Overall, we contribute to the conclusion that there is dose-dependent feedback between the immune system and the circadian clock via SA signalling.

Li et al.^32^ found that the *npr1-1* mutant displayed a larger reduction in the amplitude of *CCA1* and *LHY* upon SA treatment, suggesting that NPR1 acts as an antagonist of the effects of SA on the clock. Zhou et al.^30^ also observed that NPR1 was essential for the effect of SA on the clock as the *npr1-1* mutant did not exhibit the SA-induced increase in amplitude of *TOC1* rhythms. We found that the SA receptor NPR1 was crucial for the SA-induced period shortening. The amplitude effects we observed were not consistent between replicates and experiments, and therefore we chose not to report on them here. Significantly, we also found that the *npr1-1* mutant displayed a longer period for both *CCA1* and *TOC1* expression in uninduced conditions. This novel finding indicates that basal NPR1 levels, as well as SA-induced NPR1, modulates the circadian clock. The NPR1-dependent SA alteration of circadian clock gene rhythms may function to harness that clock’s modulation of other cellular processes in order to prioritize an immune response when pathogen attack is detected.

Existing literature reports the importance of *CCA1* in launching an immune response to primary bacterial infection. Zhang et al.^31^ report that *CCA1ox* plants exhibit increased susceptibility to Pseudomonas infection compared to wild type. Conversely, it has also been observed that *CCA1* overexpression confers increased resistance to oomycete pathogen *Hyaloperonospora arabidopsidis (Hpa)* while the *cca1* mutant displayed increased susceptibility^31,40^.

Our study aimed to expand upon this knowledge of *CCA1*’s role in immunity by investigating the potential involvement of core clock genes in SA-induced immunity. We demonstrate the importance of the circadian clock in launching an effective immune response and expand upon the critical role of *CCA1* in this coordination. We found that SA-induced *PR1* transcript levels were higher during the subjective night, though not rhythmic. Plants overexpressing *CCA1* exhibited a larger difference in SA-induced *PR1* transcript levels between dawn and dusk compared to wild type. Conversely, plants lacking *CCA1* did not display the temporal difference in SA-induced *PR1* transcript levels. This suggests that clock gene *CCA1* is required for the temporal difference in SA-induced *PR1* transcript levels. However, as *cca1lhy* exhibits a shorter period under constant conditions^31,41^, it is possible that we missed the peak/trough in SA-induced *PR1* transcript levels at the time points used in our experiment.

In addition, we observed that *CCA1* is also crucial for the downstream function of establishing SA-induced resistance to Pseudomonas. We found that priming with SA did not induce resistance in *CCA1ox* or *cca1lhy* as it did in *Col-0*, suggesting that *CCA1* is crucial for the resistance response following SA treatment. We did not observe increased susceptibility to *Pseudomonas* in *CCA1ox* as reported by Zhang et al.^31^. *CCA1* is a key morning clock gene and master regulator of circadian rhythms. Plants overexpressing *CCA1* have an arrhythmic clock under LD and LL conditions^36^. *TOC1ox* (also arrhythmic^37^) displayed SA-induced resistance to *Pseudomonas*, suggesting that the lack of SA-induced resistance in *CCA1ox* is not due to overall clock arrhythmia. As the *cca1lhy* mutant also did not display an SA-induced response, regulated *CCA1* expression levels appear to be important for establishing SA-induced resistance. We did not observe increased sensitivity to SA treatment in *toc1-101* mutant plants compared to *Col-0* as reported by Zhou et al.^30^. This could be due to differences in the timing of treatment and inoculation, which were carried out at subjective dusk (ZT12) by Zhou et al.^30^ but at subjective dawn (ZT0) in our experiments. Overall, these findings indicate that the function of *CCA1* as transcription factor outside of its role as a core clock component is responsible for its effect on the SA-induced immune response.

From our study, it is evident that SA utilizes NPR1 for modulating rhythmicity of core clock components. NPR1 is crucial for many immune processes downstream of SA. The *npr1-1* mutant displays increased susceptibility to infection, a lack of PR gene expression upon infection/SA treatment and an inability to establish resistance following priming with SA^20,21^. As we have shown that vice versa the circadian clock is also involved in regulating SA induced PR gene expression and resistance to *Pseudomonas*, it would be interesting to investigate whether NPR1 plays a role in this interaction, potentially facilitating crosstalk between the circadian clock and the immune system in both directions. Additionally, as redox regulation of NPR1 conformation and localization is a key step in its activation^42,43^, daily redox rhythms^44,45^ may control the interplay between the circadian clock and NPR1.

Our study provides evidence of the reciprocal regulation between the circadian clock and SA signalling. We show the importance of NPR1 in this interaction and that clock gene *CCA1* is key for regulation of SA-induced immune responses. The findings presented here further our understanding of the interactions between the circadian clock and the immune system and how they work together to produce an effective immune response. Continued study of the systems controlling plant immunity is essential to prioritize plant health and thus food security.

## Methods

### Plant materials and growth conditions

*Arabidopsis thaliana* were grown on soil for 3-4 weeks, unless otherwise stated, in growth cabinets (Percival Scientific Inc.) set at constant 21 °C with a light intensity of 70-100 µmoles m^-2^ s^-1^ (Fusion 18 W T8 2 ft Triphosphor Fluorescent Tube 4000 K) under 12 h light/12 h dark. All wild type and mutant plants were from the Col-0 genetic background and were described previously: *npr1-1*^21^; *CCA1ox*^36^; *TOC1ox*^37^; *cca1lhy*^31^; *toc1-101*^38^; *CCA1pro:LUC*^46^ and *TOC1pro:LUC*^47^. Experiments involving NPR1 use the single mutant allele *npr1-1*, as the presence of second-site mutation is unlikely due to extensive backcrossing^20,21^ and whole genome sequencing^48^.

*CCA1pro:LUC npr1-1 and TOC1pro:LUC npr1-1* plants were made by crossing *CCA1pro:LUC* with *npr1-1* and *TOC1pro:LUC* with *npr1-1* respectively. To obtain homozygous lines of *npr1-1* plants, individuals in the F1 and F2 generations were evaluated by polymerase chain reaction (PCR) genotyping and restriction fragment length polymorphism (RFLP) analysis. DNA was extracted using a one-step protocol previously described in Edwards et al.^49^. For PCR amplification, 0.5 μl of the DNA extract was added to 10 μl of 5X GoTaq Reaction Buffer (Promega) and 1 μl of each primer (10 μM, NPR1 F: CTCGAATGTACATAAGGC, NPR1 R: CGGTTCTACCTTCCAAAG^20^), to a total volume of 20 μl. PCR conditions were 95 °C for 5 min, 35 cycles of 95 °C for 30 s, 55 °C for 30 s and 72 °C for 1 min, followed by 72 °C for 5 min. PCR products were digested with 0.4 μl of restriction enzyme NlaIII for 2 h at 37 °C (New England Biolabs). Digested PCR products were run on 2% agarose gels with SYBR Safe (Invitrogen) at 100 V and were imaged using the Odyssey FC imaging system (LI-COR).

Homozygous *npr1-1* lines were tested for homozygosity of the luciferase transgene (either *CCA1pro:LUC* or *TOC1pro:LUC*) in F3. A minimum of 50 seeds of each line were visualized for luminescence by sowing one seed per well, on top of 200 μl 0.5 MS in black 96-well plates (Greiner bio-one, 655075). Seedlings were grown for 10 days in an incubator (Sanyo) under long-day conditions. 20 μl of 1 mM D-luciferin (Biosynth AG) was added to each well and luminescence was visualized using the ALLIGATOR luminescence imaging system by exposing the camera for 8 min. Ratios of luminescence were assessed: homozygous lines which displayed luminescence in all seedlings were used for subsequent experiments.

### Bioluminescence assay of leaf discs

*CCA1pro:LUC, TOC1pro:LUC, CCA1pro:LUC npr1-1* and *TOC1pro:LUC npr1-1* were grown on soil under LD conditions (see plant materials and methods). At 3-4 weeks old, leaf disks were taken from leaves 5-6 and placed in the wells of a white, flat-bottom 96-well plates (Greiner bio-one, 655075) with the adaxial side up. Each well also contained 200 µl of filter sterilized imaging solution: 0.5 MS pH 7.5, 50 µg/ml ampicillin, 1.5 mM D-luciferin and SA/H_2_O to the desired final concentration. The plate was sealed with a clear, gas permeable lid (4titude, 4ti-0516/96). Luminescence was measured by a LB942 Tristar2 plate reader (Berthold Technologies Ltd) every 50 min for 3 s per well. Leaf disks were kept under continuous red (630 nm) and blue (470 nm) LED light at 17.5 µmoles m^−2^ s^−1^ each at 20–21 °C. Results were analysed using GraphPad Prism and Biodare2^50^ and period was estimated using the Fast Fourier Transform-Non-Linear Least Squares (FFT-NLLS) function. Unless otherwise specified, luminescence data are displayed as residual luminescence, wherein a polynomial was fitted to the raw luminescence data and the residual calculated from this using GraphPad Prism.

### SA treatment for quantitative PCR in wildtype over 48 h

Wild-type plants (*Col-0*) were grown on MS agar plates under 12 h light/12 h dark conditions at 21 °C with a light intensity of 70–100 µmoles m^−2^ s^−1^ (Fusion 18 W T8 2 ft Triphosphor Fluorescent Tube 4000 K). After 14 days, the seedlings were released into constant light (LL). Plates were removed from the growth cabinet and sprayed with 1 mM SA or mock (H_2_O) at 3-h intervals beginning at LL24 and finishing at LL72. After spraying, the plates were put into separate growth cabinets from the untreated plates. The seedlings were harvested and frozen in liquid nitrogen 6 h after treatment.

### SA treatment for quantitative PCR in clock mutants

Wild type (*Col-0*), *CCA1ox* and *cca1lhy* plants were grown on MS agar plates under 12 h light/12 h dark conditions at 21 °C with a light intensity of 70–100 µmoles m^−2^ s^−1^ (Fusion 18 W T8 2 ft Triphosphor Fluorescent Tube 4000 K). Plates were placed under split entrainment conditions in separate growth cabinets, aligning the time of dawn and dusk between the two cabinets. After 14 days, the seedlings were released into LL. Plates were sprayed with 1 mM SA or mock (H2O) at subjective dawn (LL24) or dusk (LL36). The seedlings were harvested and frozen in liquid nitrogen 6 h after treatment.

### RNA extraction and cDNA synthesis

Seedlings were manually ground to a fine powder in liquid nitrogen, before being homogenized in RNA extraction buffer (100 mM LiCl, 100 mM Tris pH 8, 10 mM EDTA, 1% SDS). An equal volume of phenol/chloroform/isoamyl alcohol (25:24:1) was added, followed by votexing and centrifuging at 13,000 rpm for 5 min. The aqueous phase was transferred to a tube containing an equal volume of chloroform/isoamyl alcohol (24:1), followed by vortexing and centrifuging at 13,000 rpm for 5 min. This step was repeated before the aqueous phase was transferred to a tube containing 1/3 vol of 8 M LiCl and incubated overnight at 4 °C. The samples were centrifuged at 13,000 rpm at 4 °C for 15 min. The supernatant was removed and the pellet was washed twice in ice-cold (−20 °C) 70% ethanol. The pellet was then dissolved in 400 µl H_2_O for 30 min on ice. 40 µl of NaAc (pH 5.3) and 1 ml of ice-cold 96% ethanol was added and the solution was incubated for at least 1 h at −20 °C. The tubes were centrifuged at 13,000 rpm for 15 min at 4 °C and the pellet was washed twice with ice-cold 70% ethanol. The final pellet was dissolved in 50 µl H_2_O. The resulting RNA was quantified using a NanoDrop spectrophotometer and diluted to standardize the concentrations across all samples. SuperScript II reverse transcriptase (Invitrogen) was used to perform reverse transcription according to the manufacturer’s instructions.

### Quantitative PCR

For SA-induced transcript levels in wild-type over 48 h (Fig. 4 and Supplementary Fig. 4), cDNA was diluted 20-fold and qPCR was performed in a 5 µl reaction using SYBR Green and gene specific primers on a QuantStudio 5 PCR machine.

For SA-induced transcript levels in clock mutants at subjective dawn and dusk (Fig. 5), cDNA was diluted 20-fold and qPCR was performed in a 10 µl reaction using SYBR Green and gene specific primers on a StepOne Plus Real Time PCR machine.

### Circadian rhythmicity analysis of transcript levels

SA-induced *PR1* transcript levels in wild type over 48 h (Fig. 4 and Supplementary Fig. 4) were assessed for circadian rhythmicity using the JTK_cycle algorithm with empirical calculation of *p*-values (eJTK) in BioDare2^35,50^. The mean transcript levels of the two biological replicates were used for analysis with parameters set as eJTK Classic, linear detrending of the input data and p < 0.05.

### Pseudomonas disease assay

Col-0, *CCA1ox, cca1lhy, TOC1ox* and *toc1-101* were grown on soil under LD conditions (see plant materials and methods). At 3.5 weeks old, plants were released into LL. At subjective dawn (LL24), plants were sprayed with 1 mM SA or mock (water). At LL48, 2 leaves (leaves 4-6) were pressure infiltrated with *Pseudomonas syringae pv. maculicola (Psm)* ES4326 at OD_600_ = 0.005, using a 1 ml syringe. The bacteria were left to infect the plants for 3 days, before leaf disks were harvested from the infiltrated leaves. The leaf disks were homogenized and diluted in 10 mM MgSO_4_, then plated onto LB plates (10 mM MgSO_4_ and 50 µg/ml streptomycin). Plates were incubated for 2 days at 28 °C, before calculating colony forming units (CFU) per leaf disk.

## Supplementary information

Supplementary Information

## Data Availability

Data sharing is not applicable to this article as no datasets were generated or analysed during the current study. Raw luminescence data can obtained from the corresponding author upon reasonable request.
