# Supplementary material for: Crosstalk between salicylic acid signalling and the circadian clock promotes an effective immune response in plants
Source: NPJ Biol Timing Sleep. 2024 Sep 2;1:6. doi: 10.1038/s44323-024-00006-0 (PMC12449264; doi:10.1038/s44323-024-00006-0)
Supplement: Supplementary file 1 — Supplementary Information [file 44323_2024_6_MOESM1_ESM.pdf]

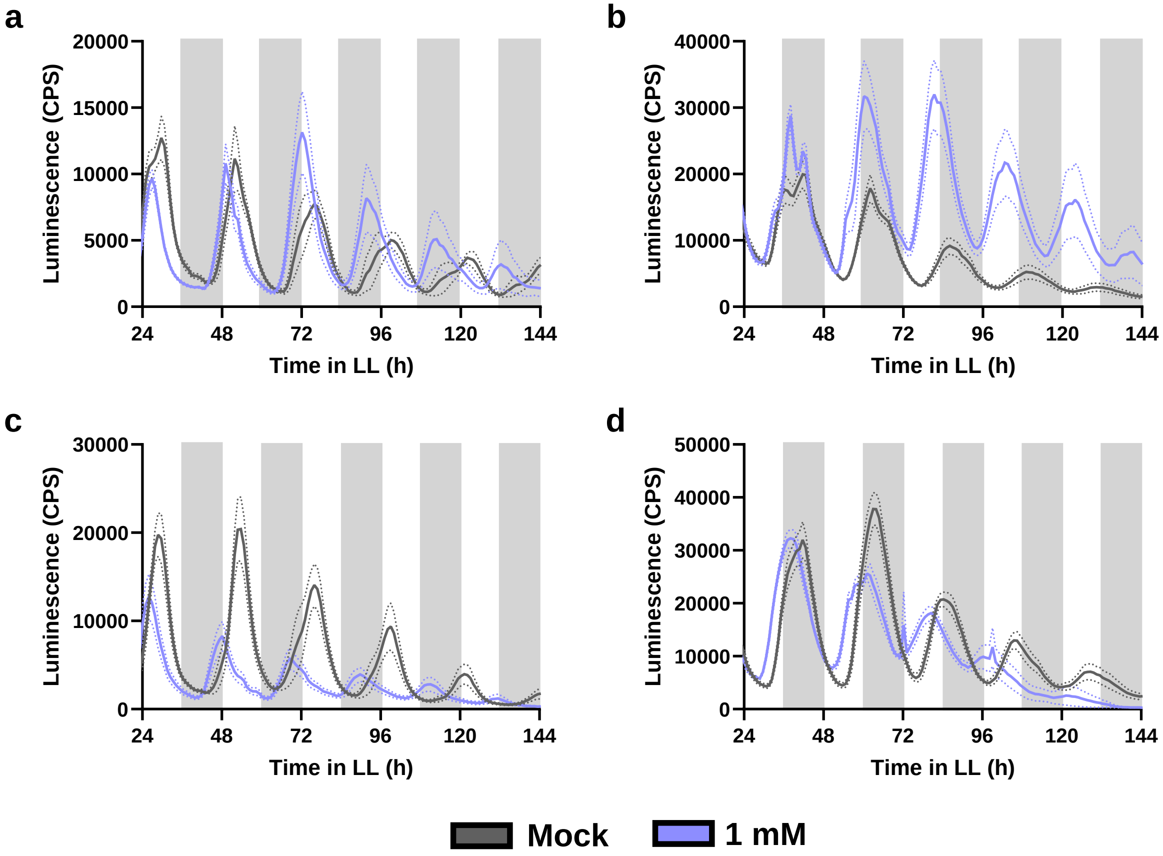

Supplementary Figure 1. Continuous SA treatment shortens the period of the circadian clock. Promoter activity of *CCA1* (a, c) and *TOC1* (b, d) was observed by measuring luminescence in *CCA1pro:LUC* and *TOC1pro:LUC* leaf disks, respectively. Leaf disks were treated with 1 mM SA (blue) or mock treated with water (grey). These raw luminescence data are the same data displayed in residual luminescence in Figure 1 (a, b) and an independent repeat of the same experiment (c, d). Error bars indicate mean ± SEM (n = 7). Data are representative of 3 independent repeats.

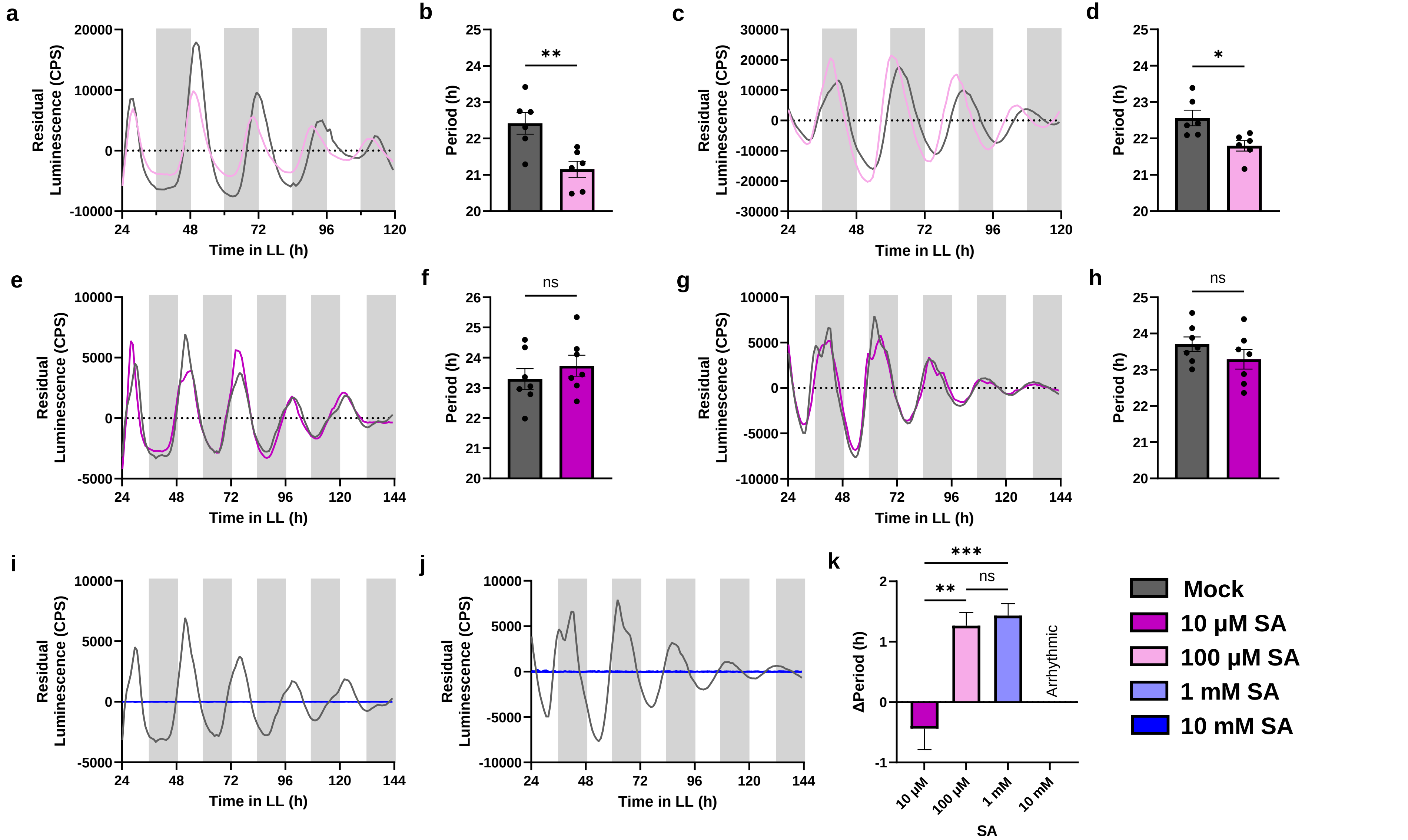

Supplementary Figure 2. SA-induced period shortening is dose dependent. Promoter activity of *CCA1* (a, e, i) and *TOC1* (c, g, j) was observed by measuring luminescence in *CCA1pro:LUC* and *TOC1pro:LUC* leaf disks, respectively. Leaf disks were treated with 100 µM SA (a, c), 10 µM SA (e, g), 10 mM SA (i, j) or mock (water). The mean period (b, d, f, h) was calculated from the 24 – 120 hr time window for each treatment. An unpaired t-test was performed between each mock and treated data set for *CCA1* and *TOC1*: *p <0.05, **p <0.01, ***p <0.001, ****p<0.0001, unpaired t-test. Error bars indicate mean ± SEM (n = 7). Data are from a single experiment representative of 3 independent repeats. The mean Δperiod (k) of *CCA1* was calculated from the mock and treated data sets and displayed with the results of a one-way ANOVA and Tukey's HSD Test comparing between treatments: *p <0.05, **p <0.01, ***p <0.001, ****p<0.0001, one-way ANOVA and Tukey's HSD Test. Error bars indicate ± SEM (n = 7). Data are from a single experiment representative of 3 independent repeats.

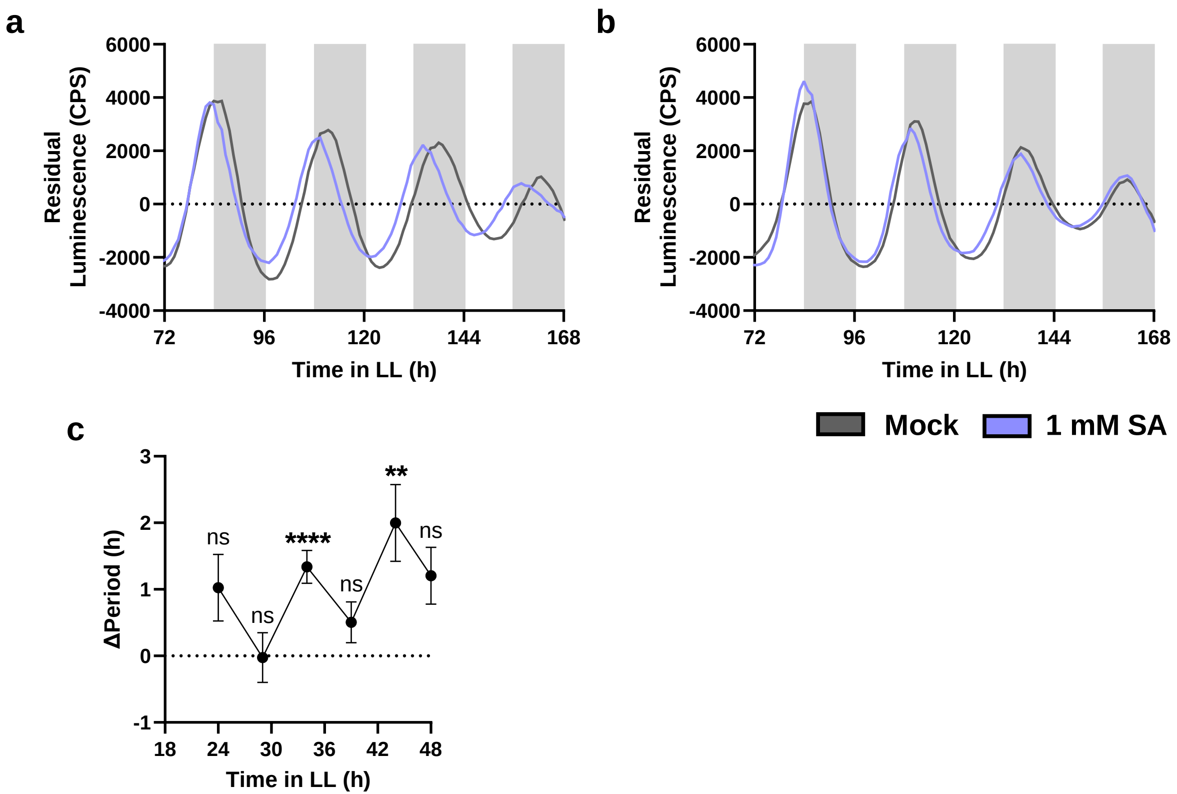

Supplementary Figure 3. Transient SA treatment shortens the period of the circadian clock when applied at different times over 24 hours. Promoter activity of *CCA1* (a, b) was observed by measuring luminescence in *CCA1pro:LUC* leaf disks. Leaf disks were treated with 1 mM SA (blue) or mock treated with water (grey) at 5 different time points over 24 hrs. The media was removed and replaced with SA-free imaging media 8 hrs post treatment. Only luminescence traces of treatments at LL34 (a) and LL39 (b) are shown here to demonstrate significant period shortening (a) and lack of period shortening (b). The mean period was calculated from the 72 – 168 hr time window and unpaired t-tests were performed between each mock and treated data set for each time point: *p <0.05, **p <0.01, ***p <0.001, ****p<0.0001, unpaired t-test. The mean Δperiod (c) was calculated from the mock and treated data sets and displayed with the results of the unpaired t-test for each time point. Error bars indicate mean ± SEM (n = 8). These data are from a single experiment representative of 2 independent repeats.

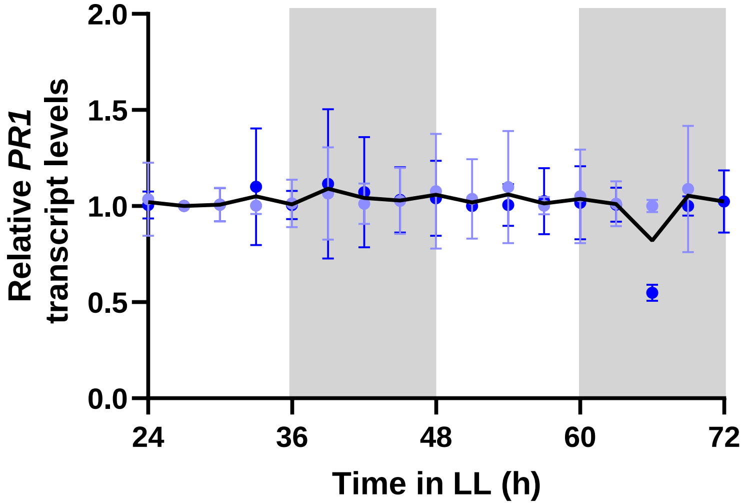

Supplementary Figure 4. Basal *PR1* transcript levels do not exhibit circadian variation. *Col-0* seedlings were grown on MS plates under 12/12 light/dark conditions for 14 days before being released into constant light (LL). Every 3 hours, plates were sprayed with water. Relative transcript levels of *PR1* were measured 6 hours post treatment. The data points are the mean ± SEM (n = 3) of quantitative PCR results from two biological replicates (light blue versus dark blue). The black line represents the mean values of the two biological replicates.

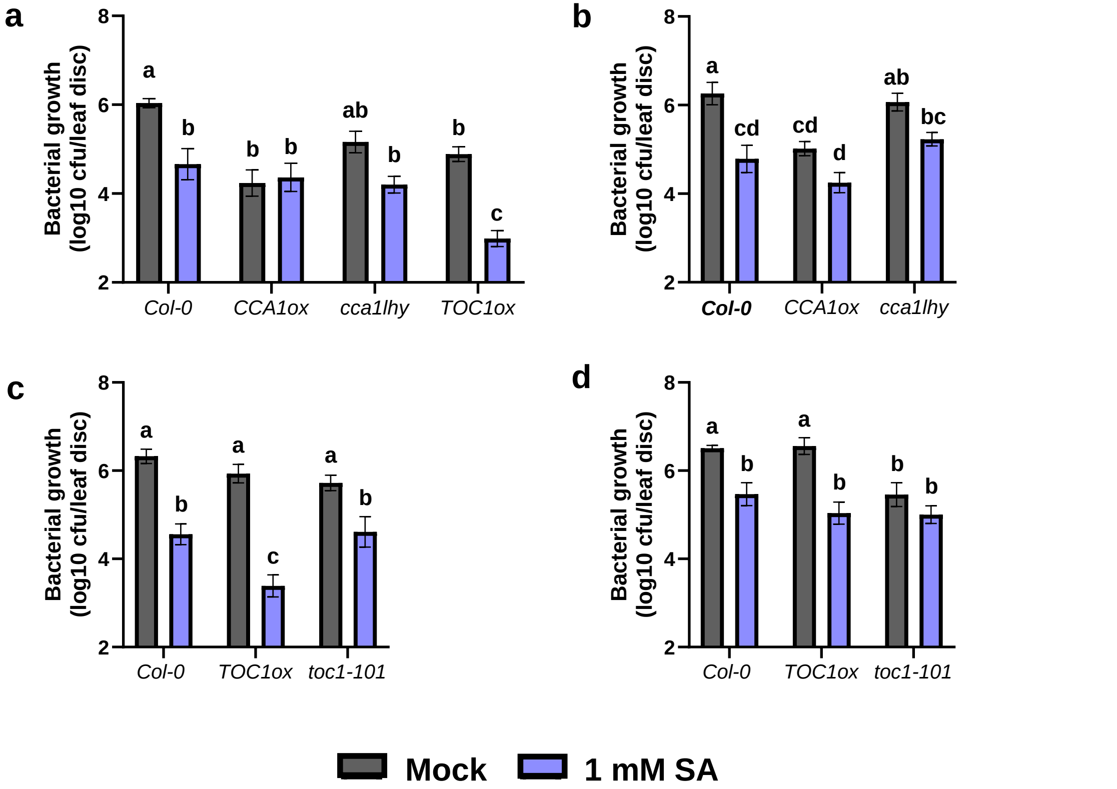

Supplementary Figure 5. *CCA1* circadian mutants do not display SA induced resistance to *Pseudomonas*. *Col-0*, *CCA1ox*, *cca1lhy*, *TOC1ox* and *toc1-101* plants were grown under 12/12 light/dark conditions for 3 weeks. At dawn on day 21 (LL0), the plants were released into constant light conditions. At LL24 the plants were sprayed with 1 mM SA or mock (water). At LL48 the plants were infected with *Pseudomonas syringae pv. maculicola (Psm)*. The level of bacterial growth was measured 3 days post infection. The infection level at 3 days post infection is expressed by colony forming units (CFU, y-axis is logarithmic) in leaf disk extracts. Error bars represent mean ± SEM (n = 8 individual leaves). A two-way ANOVA and Tukey's HSD Test were preformed to analyse the differences in bacterial growth between treatments and genotypes. The letters indicate significant differences between groups ($p < 0.05$). The data shown here are from 2 independent repeat experiments for *Col-0* versus *CCA1ox* or *cca1lhy* (a, b), 3 independent repeat experiments for *Col-0* versus *TOC1ox* (a, b, c) and 2 independent repeat experiments for *Col-0* versus *toc1-101* (c, d).
